# Acute effects of mechanical dyssynchrony on left ventricular function and coronary perfusion

**DOI:** 10.3389/fbioe.2025.1630854

**Published:** 2025-09-12

**Authors:** Jenny S. Choy, Lei Fan, Yousif Awakeem, Chenghan Cai, Farshad Raissi, Lik Chuan Lee, Ghassan S. Kassab

**Affiliations:** ^1^ California Medical Innovations Institute, Inc., San Diego, CA, United States; ^2^ Department of Mechanical Engineering, Michigan State University, East Lansing, MI, United States; ^3^ Department of Medicine, University of California San Diego, San Diego, CA, United States

**Keywords:** mechanical dyssynchrony, right ventricular pacing, left ventricular hemodynamics, left ventricular function, coronary blood flow

## Abstract

**Background:**

Patients with heart failure frequently develop mechanical dyssynchrony, which impairs ventricular function, coronary perfusion and their interactions. The underlying mechanisms, however, remain poorly understood due to numerous confounding factors. The objective of this study was to determine the acute effects of mechanical dyssynchrony on global and regional left ventricular (LV) function, coronary perfusion and their interactions based on experimental and computational approaches.

**Methods:**

Mechanical dyssynchrony was created with right ventricular apical pacing in Yorkshire domestic swine (n = 9). The heart was paced at 100 and 140 bpm and the results were compared to right atrial pacing. An inverse finite element computational framework based on an animal-specific geometry of the LV and measurements was developed to investigate the effects of mechanical dyssynchrony on LV function and its correlation with regional coronary perfusion.

**Results:**

Cardiac dyssynchrony induced significant decrease in LV pressure, volume, dP/dt_(min)_, stroke volume, ejection fraction, and regional longitudinal and circumferential strain. With mechanical dyssynchrony, passive flow decreased by 70% in the left anterior descending artery (LAD) and 67% in the left circumflex (LCX). An animal-specific inverse finite element computational model predicted that in mechanical dyssynchrony, global and regional LV contractility in the septum and LV free wall (LVFW), and myocardial work done in the septum and LVFW decreased.

**Conclusion:**

The computational model predicted reduction in global and regional contractility, and regional myocardial work done in the septum and LVFW with mechanical dyssynchrony are positively correlated with the corresponding decrease in experimentally measured regulated coronary flow in the LAD and LCX. These findings demonstrate that this interrelated mechanism between LV function and coronary flow in mechanical dyssynchrony may affect cardiac resynchronization therapy responder rate.

## Highlights

• Mechani cal dyssynchrony induced in swine via right ventricular pacing resulted in a significant decrease in left ventricular (LV) end-systolic pressure, dP/dt_(min)_, end-diastolic volume, ejection fraction and regional passive flow.
• An animal-specific inverse finite element computational model predicted that the adverse impact of mechanical dyssynchrony on regional LV function may be related to changes in regional coronary perfusion, having clinical implications in improving non-responders rate of cardiac resynchronization therapy.

## Introduction

Heart failure (HF) is a chronic disease that has been recognized as an emerging epidemic affecting approximately 1%–2% of the adult population (Schwinger, 2021), with the majority of cases resulting from damage to the myocardium due to ischemic heart disease (Ziaeian and Fonarow, 2016). In the 1990s, cardiac resynchronization therapy (CRT) was introduced for the treatment of patients with advanced HF and with evidence of electrical and mechanical dyssynchrony. Since then, CRT has shown to reduce morbidity and mortality (Boriani et al., 2015; Normand et al., 2018) as well as hospitalizations (Solo et al., 2010) by reversing many of the abnormalities observed in these patients. Approximately 30%–50% of these patients, however, do not respond to the therapy (Kirk and Kass, 2013; Lee et al., 2018), and the number of non-responders has remained consistent over time (Gorcsan, 2011).

Multiple contributing factors like the presence of myocardial scar tissue (Harb et al., 2019), left ventricular (LV) dyssynchrony (Cazeau et al., 2019), myocardial contractile reserve (Murín et al., 2015), and cellular and molecular mechanisms (Spragg et al., 2003; Chakir et al., 2008) among others, have been recognized as important elements in the identification of non-responders, which until now remains challenging. Understanding the interactions between these elements can significantly improve current clinical management of HF patients.

Among these elements, coronary perfusion has recently been suggested in several clinical studies to have prognostic significance in CRT response (Cortigiani et al., 2013). These clinical studies, however, are confounded by effects associated with possible (chronic) remodeling of the coronary vasculature and myocardium. Related acute animal studies on coronary perfusion (Prinzen et al., 1990; Ono et al., 1992; Amitzur et al., 1995; Beppu et al., 1997), on the other hand, are performed largely on canine with substantial differences in coronary anatomy compared to humans. Besides, regional contractile function has been proposed to quantify the LV function, for which the maximum gradient of LV pressure (dP/dt_(max)_) has been used (Monge Garcia et al., 2018; Fan et al., 2021a). This index can only be used as a global measurement, however, and it is also a load-dependent parameter that might be affected by preload, afterload and geometry (Wang et al., 2015). To address these limitations, the objective of the present study was to determine the effects of dyssynchrony on cardiac hemodynamics, LV function, and coronary blood flow in a swine model of RV-paced rhythm. An animal-specific inverse finite element (FE) computational modeling framework was developed to quantify the effects of dyssynchrony on regional contractility and myocardial work, providing insights synergistic with the experimental studies. This study is foundational to understand the interaction between dyssynchrony and potential ischemia.

## Methods

### Animal experiments

#### Animal preparation

All animal experiments were performed in accordance with national and local ethical guidelines, including the Guide for the Care and Use of Laboratory Animals, the Public Health Service Policy on Humane Care and Use of Laboratory Animals, and the Animal Welfare Act, and an approved California Medical Innovations Institute Institutional Animal Care and Use Committee protocol regarding the use of animals in research.

Yorkshire domestic, female swine (n = 9), with body weight of 58.9 ± 3.4 kg, were used in this study. The animals were housed at California Medical Innovations Institute–Animal Care Facilities. After overnight fasting, sedation was achieved with TKX (Telazol 10 mg/kg, Ketamine 5 mg/kg, and Xylazine 5 mg/kg, IM) and surgical anesthesia was maintained with isoflurane 1%–2%. Ventilation with 100% oxygen was provided with a respirator and maintained PCO_2_ at approximately 35–40 mmHg. Electrocardiographic leads were attached to the animal’s limbs to monitor the electrical activity of the heart. Introducer sheaths were placed in the right jugular vein to advance two pacing leads into the right atrium and the right ventricle, respectively, and the right femoral artery to access the LV and left anterior descending (LAD) and left circumflex (LCX) arteries. The animals were heparinized (100 IU/kg, IV) before further instrumentation.

#### Right atrial and right ventricular pacing

The heart was paced individually from the right atrium and right ventricle (apex) at rates of 100 and 140 bpm using temporary pacing electrode catheters (Bard Medical, Covington, GA) connected to an external pacemaker (Pace 203H, Oscor, Palm Harbor, FL, USA). These pacing heart rates (100 and 140 bpm) above each animal’s intrinsic heart rate were chosen because the His bundle was not ablated in this study. They were intended to reflect exercise conditions as our goal here was to understand the impact of mechanical dyssynchrony on coronary blood flow and its reserve. This also ensured consistency of heart rates across 9 swine and allowed direct rate comparisons, as intrinsic heart rates varied between animals. To pace from the right atrium (right atrial pacing, RAP), the dual pacing, dual sensing, dual inhibition (DDD) mode was used, and to pace from the right ventricular apical region (right ventricular pacing, RVP), the single chamber ventricular (VVI) pacing mode was used. The pacing modes were applied in a random sequence.

#### Left ventricular pressure and volume

LV pressure and volume were measured using a 5F pressure-volume catheter (Ventri-Cath 507, Millar, Inc., Houston, TX), connected to an MPVS Ultra PV loop system (Millar, Inc., Houston, TX). The catheter was advanced into the LV apex through an introducer sheath in the femoral artery using a 6F guide catheter. Pressure and volume waveforms were recorded using a data acquisition system (LabChart Pro, ADInstruments, Colorado Springs, CO). LV end-diastolic (LVEDP) and end-systolic pressures (LVESP), dP/dt_(max)_, dP/dt_(min)_, end-diastolic (LVEDV) and end-systolic volumes (LVESV) were determined and averaged over a minimum of 10 cardiac cycles using LabChart Pro.

#### Echocardiography

Transesophageal echocardiograms were obtained using an EPIQ 7C ultrasound system (Philips, Andover, MA) with an X8-2t transducer. Four-chamber two-dimensional and three-dimensional (3D) echocardiographic (ECHO) images were acquired with the animals placed in the supine position while simultaneously recording LV pressure. LVEDV, LVESV, stroke volume, ejection fraction (EF), and cardiac output were calculated offline using QLAB 10.8 (Philips Healthcare, Andover, MA). 3D ECHO images associated with the ES time point were postprocessed using TomTec Arena (2014–2020) Imaging Systems GmbH (Philips Healthcare, Andover, MA) to segment the LV endocardial and epicardial surfaces, as well as regional circumferential and longitudinal strain waveforms.

#### LV dyssynchrony index

Strain-based systolic dyssynchrony index (SDI) was calculated from 3D echocardiographic images using TomTec Imaging Systems GmbH (Philips Healthcare, Andover, MA). SDI is defined as the standard deviation of time to peak segmental strain over 16 LV segments from longitudinal, circumferential, and radial strain. Systolic dyssynchrony index provides a quantitative measure of mechanical dyssynchrony, which reflects the functional consequences of RV pacing-induced activation and has been widely used as a surrogate marker for mechanical dyssynchrony. The inclusion of SDI offers valuable insights into the degree of RV pacing-induced mechanical dyssynchrony.

#### Coronary flow rate

The chest was opened through a midline sternotomy and an incision was made in the pericardial sac with the creation of a sling to support the heart. The LAD and LCX arteries were carefully dissected free from their surrounding tissue in their proximal regions and a 3 mm flow probe connected to a flow meter (Transonic, Ithaca, NY) was placed around the coronary arteries to measure the mean flow rate. The regulated coronary flow rate was measured under resting conditions with autoregulation, whereas the passive coronary flow rate was measured under adenosine-induced vasodilation conditions.

#### Coronary flow reserve

Coronary flow reserve (CFR) was measured in both the LAD and LCX arteries (proximal regions) using a pressure/flow guide wire (ComboWire XT, Philips Healthcare, Andover, MA) connected to a ComboMap system (Philips Healthcare, Andover, MA) through the patient interface module. For induction of hyperemia, 120 μg of intracoronary adenosine was administered as a bolus injection. The CFR index was monitored to ensure the vessel was under fully-dilated condition (CFR = 1).

Coronary flow reserve (CFR) can also be calculated as the ratio of passive to regulated coronary flow rate measured using flow probes. CFR determined from coronary flow rates measured using flow probes were analyzed to quantify the effects of RVP on CFR, which is consistent with the approach used to assess the effects of RVP on coronary flow rates.

### Computational modeling

#### Constitutive modeling of the LV

Details of the inverse finite element (FE) modeling framework for estimating regional contractility from 3D ECHO images can be found in our previous work (Zhang et al., 2024). Briefly, a finite element (FE) mesh was generated in the LV wall defined by the endocardial and epicardial surfaces segmented from the 3D ECHO images. Therefore, in this study, 9 animal-specific LV geometries were used in the computational simulations. The functional relationship between pressure and volume in the LV was obtained by minimizing a functional consisting of a myocardial tissue strain energy function, active stress and terms associated with enforcing constraints on myocardial tissue incompressibility, zero-mean rigid body translation and rotation, and cavity volume (Fan et al., 2021a; Zhang et al., 2024; Fan et al., 2021b; Fan et al., 2023; Mojumder et al., 2023). Additionally, the LV base was constrained from moving out of the plane.

#### Estimation of active parameter

Parameters associated with passive mechanics are first estimated in the inverse FE modeling framework following which, the global and regional active stress parameter $T_{\max}$ are then estimated by solving a PDE-constrained optimization problem (Finsberg et al., 2018; Finsberg et al., 2019). The cost function in the optimization problem describes the mismatch between the simulation and measured data. The minimization problem is stated as:

$$\text{Minimize }J\left(\left(U,p\right),T_{\max}\;\right)\text{ subject to }\delta \Pi \left(U,p\right)=0$$ (1)

In Equation 1, J is the objective function that is minimized, depending on the state variable displacement U and hydrostatic pressure p , as well as the (control) global and regional active stress parameter $T_{\max}$ that reflects myocardial contractility. The state variables also depend on the control parameters $\left(U,p\right)=\left(U\left(T_{\max}\right),p\left(T_{\max}\right)\right)$ . The constraint $\delta \Pi \left(U,p\right)=0$ in the optimization problem is the Euler-Lagrange equation or the weak formulation of the mechanical equilibrium governing equations (Finsberg et al., 2018).

At each time point i , the global and regional active stress parameter ${T_{\max}}^{i}$ is estimated based on the measured cavity volume $V_{LV}^{i}$ by minimizing the cost function:

$$J\left(\left(U^{i},p^{i}\right),{T_{\max}}^{i}\right)={\left(\frac{P_{LV}^{i}-{\tilde{P}}_{LV}^{i}}{P_{LV}^{i}}\right)}^{2}+{\left(\frac{E_{cc}^{i}-{\tilde{E}}_{cc}^{i}}{E_{cc}^{i}}\right)}^{2}+{\left(\frac{E_{ll}^{i}-{\tilde{E}}_{ll}^{i}}{E_{ll}^{i}}\right)}^{2}.$$ (2)

The cost function in Equation 2 defines the mismatch between simulated cavity pressure ${\tilde{P}}_{LV}^{i}$ , circumferential strain ${\tilde{E}}_{cc}^{i}$ and longitudinal strain ${\tilde{E}}_{ll}^{i}$ , and measured cavity pressure $P_{LV}^{i}$ , circumferential strain $E_{cc}^{i}$ and longitudinal strain $E_{ll}^{i}$ , at time point i , respectively. Based on this cost function, ${{\;T}_{\max}}^{i}$ is estimated at each discrete time point i to obtain its corresponding waveform $T_{\max}\left(t\right)$ over a cardiac cycle.

### Statistical analysis

All statistical analyses were performed using SigmaStat 3.5 (Systat Software, Point Richmond, CA). The data were expressed as mean ± standard deviation (SD), unless otherwise specified. The differences between the various parameters and groups were evaluated using analysis of variance (ANOVA) and paired Student’s t-test. To correct for multiple pairwise comparisons, the Bonferroni correction method was applied. The differences were considered significant at *p* < 0.05. These statistical analyses were designed to directly test the specified hypotheses on the effects of RVP as compared to RAP within each animal, and differences at two pacing rates (100 bpm and 140 bpm) and across microvascular territories (LAD and LCX) but not to evaluate all possible higher-order interactions. For this purpose, a within-animal ANOVA followed by paired t-tests provided an appropriate and statistically valid framework, enabling direct within-animal comparisons without overfitting given a limited number of animals.

### Post-processing of results

Results from the computational framework were obtained from each simulation case. Following Kerckhoffs et al. (2005), Walmsley et al. (2015), local work density in a cardiac cycle is given by the area of the myofiber stress-strain loop, namely:

$$W_{f}=\int_{cardiac\;cycle}S_{ff}.dE_{ff},$$ (3)

where ${\;S}_{ff}$ and $E_{ff}$ are PK2 fiber stress and Green-Lagrange fiber strain, respectively. Because active stress in Equation 3 is prescribed to develop only in the myofiber direction, we have considered only work in that direction, and have neglected work associated with other components of the stress and strain tensor as considered in other studies (Gsell et al., 2018). The local Green-Lagrange fiber strain is defined in Equation 4 as:

$$E_{ff}=\frac{1}{2}\left(e_{f}\cdot C\cdot e_{f}-1\right),$$ (4)

where C is the right Cauchy-Green deformation tensor and $e_{f}$ is the unit vector in the myofiber direction in the reference configuration. We note that $W_{f}$ represents only the local mechanical work density and does not consider basal metabolism and other chemical energies. The local myofiber stress-strain loop area represents the net work performed by cells locally in the tissue (Wang et al., 2012). As such, there is a basis for using area of the myofiber stress-strain loop as an index for local work density performed by the cell.

## Results

The mean values corresponding to the hemodynamics and LV function changes with RAP and RVP are summarized on Table 1. Pairwise changes of the values between RAP and RVP are described below.

**TABLE 1:** Changes in left ventricular function and coronary flow during right atrial and right ventricular pacing. BL denotes baseline that is spontaneous condition in the table.

Heart Rhythem											
Variables	Spontaneous	RAP		RVP			RAP		RVP		
	60-100 bpm	100 bpm	p-values compared to BL	100 bpm	p-values compared to BL	p-values RAP vs RVP	140 bpm	p-values compared to BL	140 bpm	p-values compared to BL	p-values RAP vs RVP
LVEDP (mmHg)	11.3 ± 4.1	12.9 ± 4.2	0.41	8.5 ± 3.1	0.133	0.02	13.8 ± 4.7	0.24	7.3 ± 4.2	0.06	0.01
LVESP (mmHg)	82.5 ± 9.1	96.4 ± 12.8	0.02	70.8 ± 15.7	0.07	0.002	96.1 ± 12.9	0.02	60.4 ± 19.9	0.01	0.001
Max LV dP/dt (mmHg/s)	1337.8 ± 208.0	1265.8 ± 193.24	0.46	1267.6 ± 153.0	0.428	0.983	1581.1 ± 281.9	0.06	1167.8 ± 369.7	0.251	0.02
Min LV dP/dt (mmHg/s)	-1903.3 ± 137.7	-2055.9 ± 239.0	0.12	-1655.7 ± 440.4	0.14	0.03	-2109.8 ± 277.9	0.07	-1178.7 ± 452.3	0.001	0.0001
LVEDV (mL)	60.4 ± 7.9	60.3 ± 8.1	0.97	45.6 ± 6.0	0.001	0.001	53.1 ± 7.4	0.07	41.8 ± 11.5	0.003	0.04
LVESV (mL)	26.3 ± 5.7	29.6 ± 5.9	0.27	25.0 ± 7.2	0.698	0.186	28.6 ± 7.9	0.52	24.4 ± 8.8	0.609	0.329
LVSV (mL)	34.1 ± 7.2	30.7 ± 4.8	0.28	20.6 ± 5.0	0.001	0.001	24.5 ± 4.7	0.01	17.4 ± 4.5	0.0001	0.01
LVEF (%)	56.3 ± 7.8	51.1 ± 6.0	0.16	45.7 ± 13.1	0.07	0.318	46.6 ± 9.8	0.05	43.5 ± 10.5	0.02	0.544
CO (mL/min)	2646.8 ± 672.5	3067.5 ± 477.9	0.17	2057.6 ± 498.4	0.07	0.001	3424.9 ± 662.1	0.04	2439.3 ± 626.6	0.534	0.01
Reg LAD Flow Rate (mL/min)	22.7 ± 8.1	27.6 ± 6.5	0.22	20.0 ± 7.9	0.48	0.05	26.5 ± 6.3	0.32	20.8 ± 5.7	0.53	0.08
Pas LAD Flow Rate (mL/min)	92.6 ± 46.2	89.6 ± 36.9	0.02	56.4 ± 21.0	0.17	0.13	81.4 ± 17.8	0.64	27.4 ± 6.8	0.03	0.001
Reg LCX Flow Rate (mL/min)	18.9 ± 8.3	18.7 ± 4.0	0.96	18.5 ± 6.1	0.86	0.87	20.4 ± 3.6	0.55	16.8 ± 4.8	0.66	0.89
Pas LCX Flow Rate (mL/min)	60.8 ± 54.9	56.3 ± 31.7	0.12	38.8 ± 23.3	0.57	0.49	51.2 ± 14.3	0.8	20.1 ± 0.6	0.33	0.06
LAD Flow Reserve	2.2 ± 1.4	2.2 ± 1.4	1	2.0 ± 1.0	0.79	0.79	2.0 ± 1.0	0.84	1.8 ± 0.7	0.6	0.68
LCX Flow Reserve	3.2 ± 1.1	3.1 ± 1.1	0.42	2.1 ± 0.4	0.32	0.65	2.1 ± 0.9	0.25	2.2 ± 0.5	0.22	0.79

RAP = Right Atrial Pacing, RVP = Right Ventricular Pacing.

LVESP = Left Ventricular End-Systolic Pressure, EDP = End-Diastolic Pressure, dP/dt = Pressure over Time, EDV = End-Diastolic Volume, ESV = End-Systolic Volume.

SV = Stroke Volume, EF = Ejection Fraction, CO = Cardiac Output, Reg = Regulated, Pas = Passive, LAD = Left Anterior Descending Artery, LCX = Left Circumflex Artery.

### Effects of RVP on global LV function

The experimentally measured SDI with RAP was 8.6% ± 1.2% at 100 bpm (*p* < 0.05) and 8.9% ± 2.0% at 140 bpm (*p* < 0.05), whereas with RVP, SDI was 14% ± 4.8% at 100 bpm *(p* < 0.05) and 19.4% ± 6.9% at 140 bpm (*p* < 0.01). The baseline values of SDI were 5.8% ± 2.3%.

**FIGURE 1:**
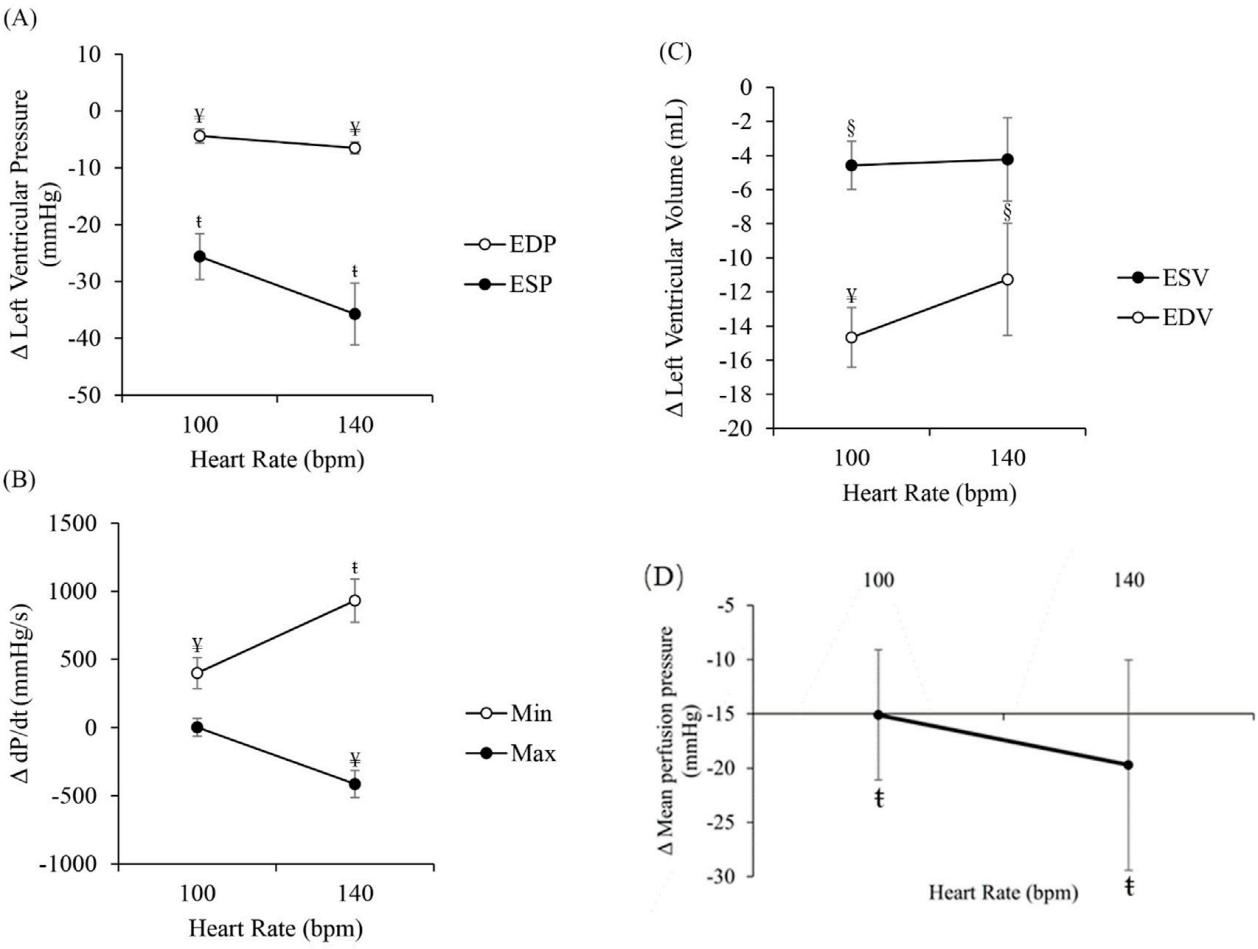
Differences between right ventricular pacing (RVP) and right atrial pacing (RAP) (RVP-RAP) in **(A)** left ventricular end-diastolic and end-systolic pressures, **(B)** left ventricular dP/dt_(max)_ and dP/dt_(min)_, **(C)** left ventricular end-systolic and end-diastolic volumes, and **(D)** mean perfusion pressure, at 100 bpm and 140 bpm. §*p* < 0.05, RAP vs. RVP ¥ *p* < 0.01, RAP vs. RVP ŧ *p* < 0.001, RAP vs. RVP.

Figure 1A shows the changes in LVEDP and LVESP between RAP and RVP. LVEDP significantly decreased with RVP (−4.4 ± 1.0 mmHg at 100 bpm, *p* < 0.01, and −6.5 ± 1.5 mmHg at 140 bpm, *p* < 0.01). Similarly, LVESP significantly decreased as well with RVP (˗25.6 ± 4.0 mmHg at 100 bpm, *p* < 0.001, and −35.7 ± 5.4 mmHg at 140 bpm, *p* < 0.001). Figure 1B shows the changes in dP/dt_(max)_ and dP/dt_(min)_ between RAP and RVP. LV dP/dt_(max)_ did not change at 100 bpm but significantly decreased at 140 bpm (−413.4 ± 297.1 mmHg/s, *p* < 0.01). The changes in dP/dt_(min)_ at 100 bpm and 140 bpm were 400.1 ± 113.6 mmHg/s (*p* < 0.01) and 931.1 ± 158.2 mmHg/s (*p* < 0.001), respectively. Figure 1C shows the changes in LVEDV and LVESV. LVEDV significantly decreased with RVP (−14.7 ± 1.8 mL at 100 bpm, *p* < 0.001, and ˗11.3 ± 3.3 mL at 140 bpm, *p* < 0.05). Similarly, LVESV significantly decreased with RVP at 100 bpm (−4.6 ± 1.4 mL, *p* < 0.05). At 140 bpm, ΔLVESV was ˗4.2 ± 2.4 mL but did not reach significance. Figure 1D shows the changes in mean perfusion pressure that is associated with the arterial pressure. Mean perfusion pressure significantly decreased with RVP (−15.1 ± 6.0 mmHg at 100 bpm, *p* < 0.001, and ˗19.7 ± 9.7 mmHg at 140 bpm, *p* < 0.001.

**FIGURE 2:**
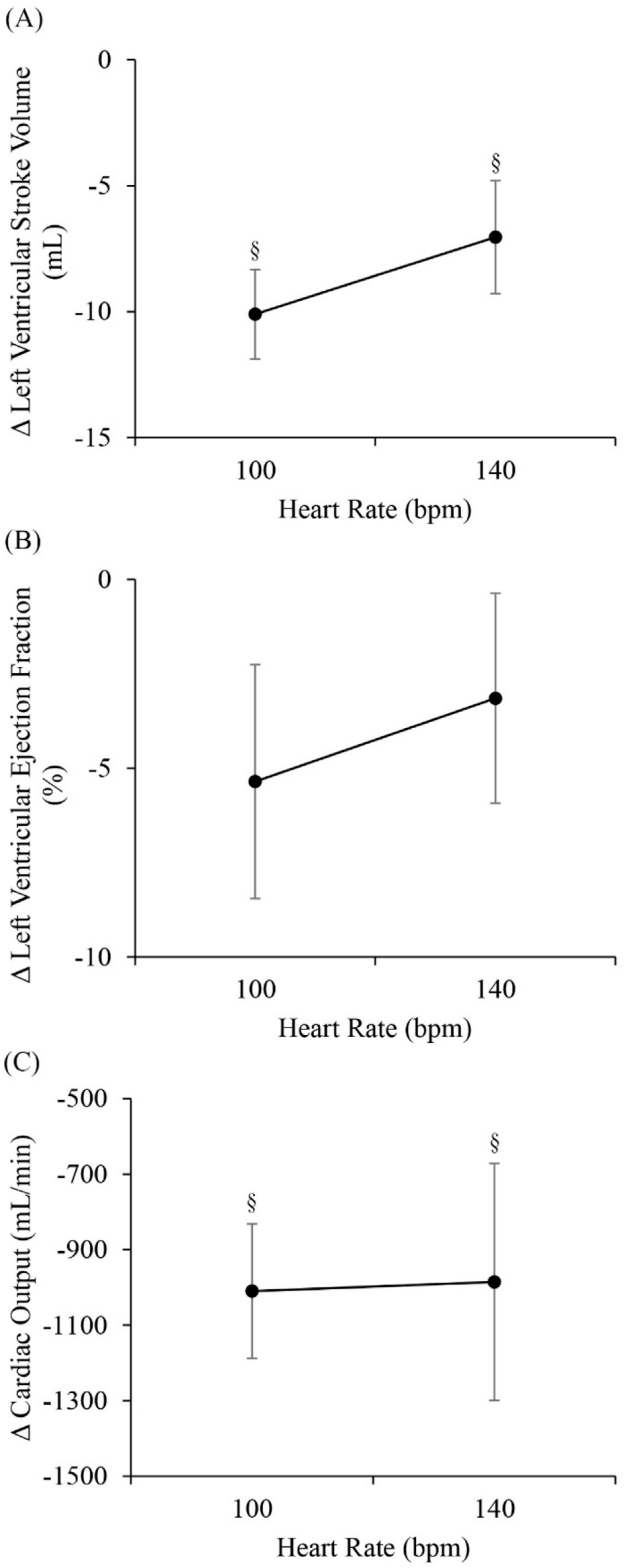
Differences between right ventricular pacing (RVP) and right atrial pacing (RAP) (RVP-RAP) in **(A)** left ventricular (LV) stroke volume, **(B)** LV ejection fraction, and **(C)** cardiac output, at 100 bpm and 140 bpm. §*p* < 0.05, RAP vs. RVP.

Figure 2 shows the changes in stroke volume, EF and cardiac output between RAP and RVP. Stroke volume significantly decreased with RVP (−10.1 ± 1.8 mL at 100 bpm, *p* < 0.05 and ˗7.0 ± 2.2 mL at 140 bpm, *p* < 0.05, Figure 2A). EF also decreased with RVP (−5.4% ± 9.3% at 100 bpm and −3.1% ± 8.3% at 140 bpm), although the reduction was not significant (Figure 2B). Cardiac output significantly decreased with RVP as well (−1009.9 ± 177.9 mL/min at 100 bpm, *p* < 0.05 and ˗985.6 ± 313.8 mL/min at 140 bpm, *p* < 0.05, Figure 2C).

**FIGURE 3:**
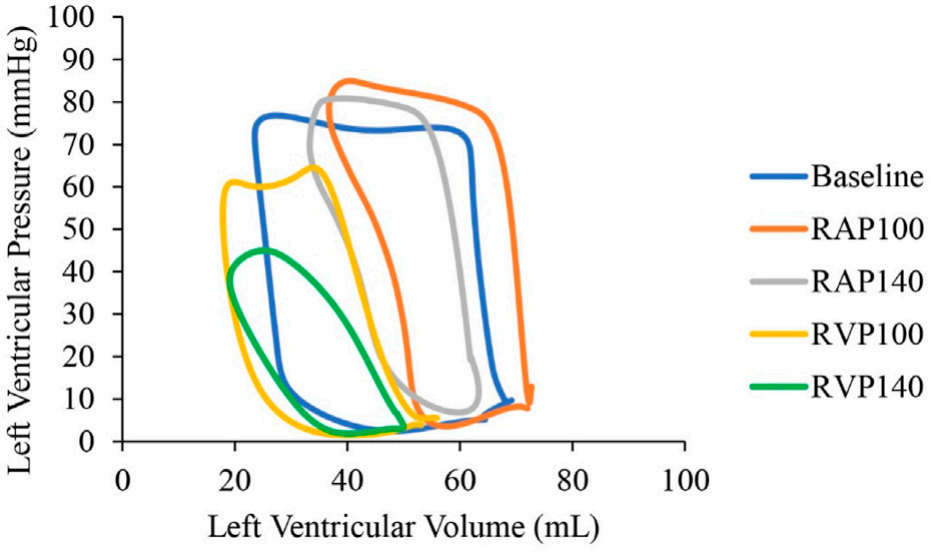
Left ventricular pressure-volume loops in a representative animal at baseline (blue), right atrial pacing (RAP) at 100 bpm (orange), RAP at 140 bpm (grey), right ventricular pacing (RVP) at 100 bpm (yellow), and RVP at 140 bpm (green).

Figure 3 shows pressure-volume loops under RAP and RVP at both 100 bpm and 140 bpm from a representative animal. It clearly shows a significant decrease in both LV pressure and volume with RVP compared to RAP at 100 bpm and 140 bpm.

### Effects of RVP on coronary perfusion

**FIGURE 4:**
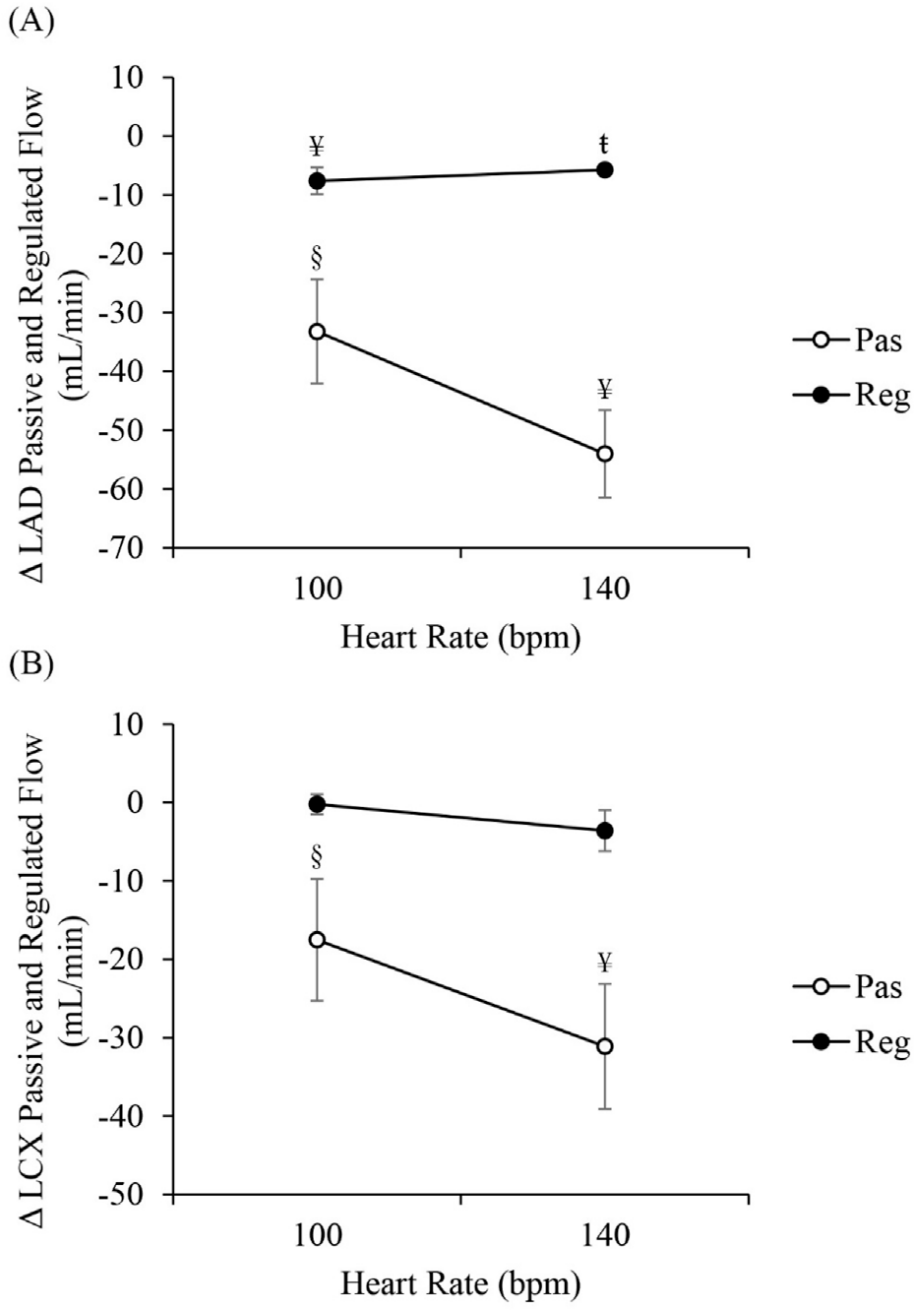
Differences between right ventricular pacing (RVP) and right atrial pacing (RAP) (RVP-RAP) in passive and regulated mean flow rate in **(A)** the left anterior descending (LAD) and **(B)** the left circumflex (LCX) arteries, at 100 bpm and 140 bpm. “Reg” denotes regulated coronary flow measured under resting condition with autoregulation. “Pas” denotes passive coronary flow measured under adenosine-induced vasodilation condition. §*p* < 0.05, RAP vs. RVP ¥ *p* < 0.01, RAP vs. RVP.

Figure 4 shows the changes in both regulated and passive mean LAD and LCX flow rates between RAP and RVP at the same heart rate. Regulated and passive mean flow rates in the LAD artery (Figure 4A) significantly decreased with RVP as compared to RAP (˗7.6 ± 2.3 mL/min, *p* < 0.01 and −33.2 ± 8.9 mL/min, *p* < 0.05 at 100 bpm, respectively, whereas at 140bpm, the change in regulated flow was −5.7 ± 0.8 mL/min, *p* < 0.001, and in passive flow −54.0 ± 7.4 mL/min, *p* < 0.01). In contrast, regulated flow in the LCX artery (Figure 4B) slightly changed (−0.2 ± 1.3 mL/min at 100 bpm and −3.6 ± 2.6 mL/min at 140 bpm). Passive flow rate, however, significantly decreased with RVP as compared to RAP (−17.5 ± 7.8 mL/min at 100 bpm, *p* < 0.05 and −31.1 ± 8.0 mL/min at 140 bpm, *p* < 0.01). Flow reduction in the LAD and LCX between RVP and RAP increased with increasing heart rate, so we expect this difference to be smaller at resting heart rate. The ratio of passive coronary flow rate to mean perfusion pressure was analyzed. This ratio from RAP to RVP (RVP-RAP) reduced by 0.23 mL/(min · mmHg) in the LAD and 0.07 mL/(min · mmHg) in the LCX at 100 bpm, and decreased by 0.68 mL/(min · mmHg) in the LAD and 0.36 mL/(min · mmHg) in the LCX at 140 bpm. These results indicate that the reduction in passive coronary flow was primarily due to decreased mean perfusion pressure.

**FIGURE 5:**
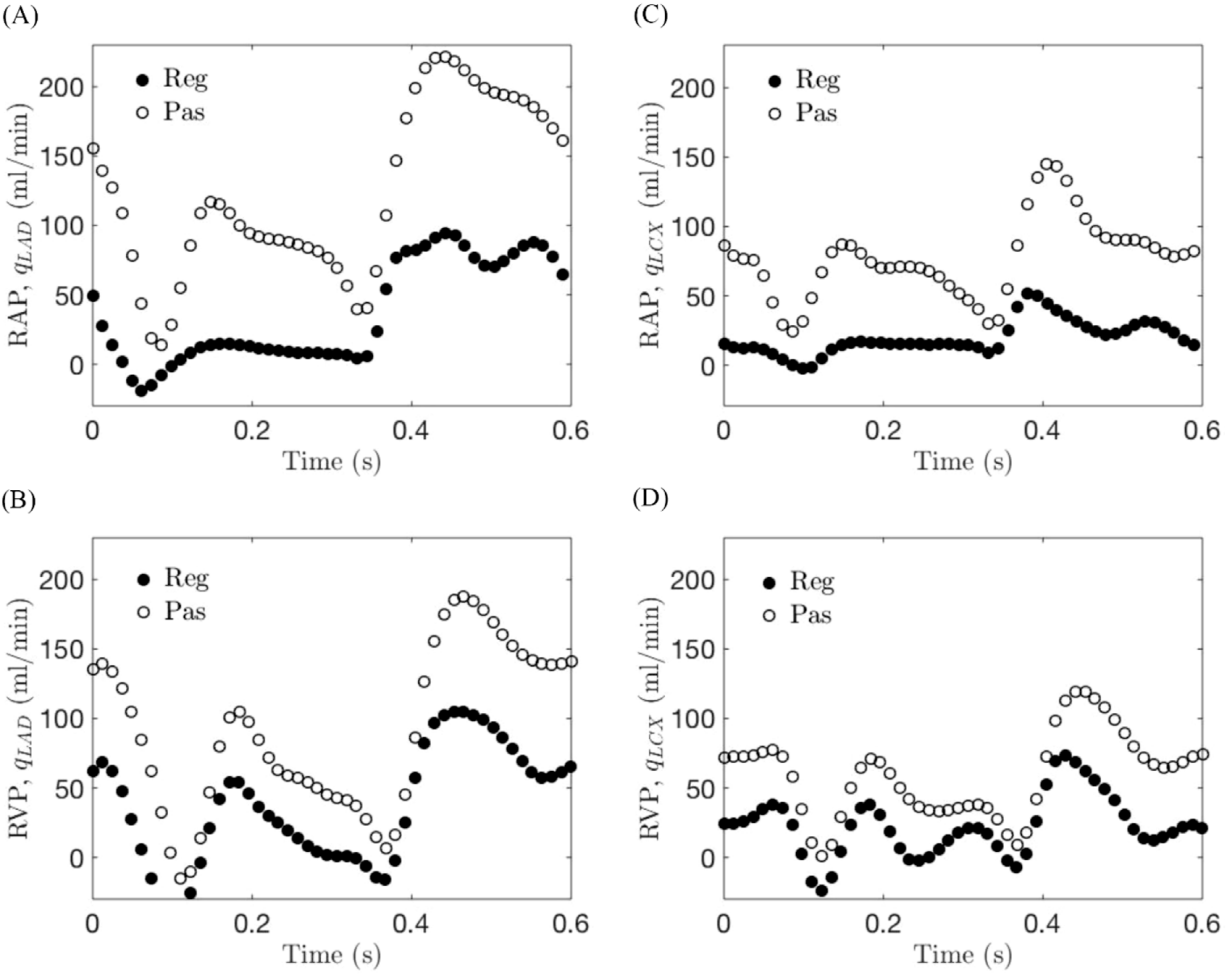
Coronary flow rate waveforms in a representative animal under regulated and passive conditions in **(A)** the left anterior descending (LAD) artery with right atrial pacing (RAP), **(B)** the LAD artery with right ventricular pacing (RVP), **(C)** the left circumflex (LCX) artery with RAP, and **(D)** the LCX artery with RVP, at pacing rate of 100 bpm. “Reg” denotes regulated coronary flow measured under resting condition with autoregulation. “Pas” denotes passive coronary flow measured under adenosine-induced vasodilation condition.

Figure 5 shows the regulated and passive waveforms of the coronary flow rates with RAP and RVP at a pacing rate of 100 bpm in a representative animal. Figures 5A,B correspond to the flow rate waveforms in the LAD artery whereas Figures 5C,D correspond to that of the LCX.

**FIGURE 6:**
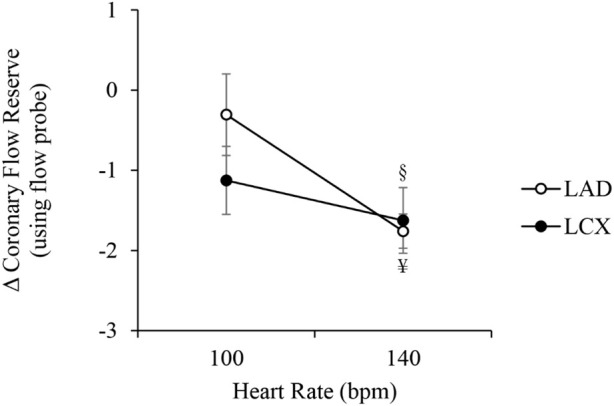
Differences between right ventricular pacing (RVP) and right atrial pacing (RAP) (RVP-RAP) in coronary flow reserve in the LAD and LCX arteries measured with flow probe, at 100 bpm and 140 bpm. “Reg” denotes regulated coronary flow measured under resting condition with autoregulation. “Pas” denotes passive coronary flow measured under adenosine-induced vasodilation condition. §*p* < 0.05, RAP vs. RVP ¥ *p* < 0.01, RAP vs. RVP.

Figure 6 shows the changes in coronary flow reserve (CFR) in the LAD and LCX arteries between RAP and RVP. The values correspond to CFR based on volumetric flow rates measured using a flow probe (Figure 6). CFR in both the LAD and LCX arteries decreased significantly at 140 bpm (−1.8 ± 0.2, *p* < 0.01 and ˗1.6 ± 0.4, *p* < 0.05, respectively). At the lowest pacing rate (100 bpm), CFR in both the LAD and LCX arteries decreased but the reduction did not reach statistical significance (−0.3 ± 0.5 and ˗1.1 ± 0.4, respectively). To provide more symmetric distributions and stabilized variance of CFR, the ratio of CFR under RVP and RAP (RVP/RAP) on the log scale with 95% confidence interval (CI) was analyzed. The ratios were −0.04 ± 0.07 (CI: [-0.15,-0.06]) in the LAD and −0.15 ± 0.07 (CI: [-0.23,-0.07]) in the LCX at 100 bpm, and −0.37 ± 0.05 (CI:[-0.44,-0.31], *p* < 0.01) in the LAD and −0.35 ± 0.17 ([-0.48,-0.21], *p* < 0.05) in the LCX at 140 bpm, demonstrating a reduction of CFR with RVP as compared to RAP.

### Effects of RVP on regional LV function: Simulation results

**FIGURE 7:**
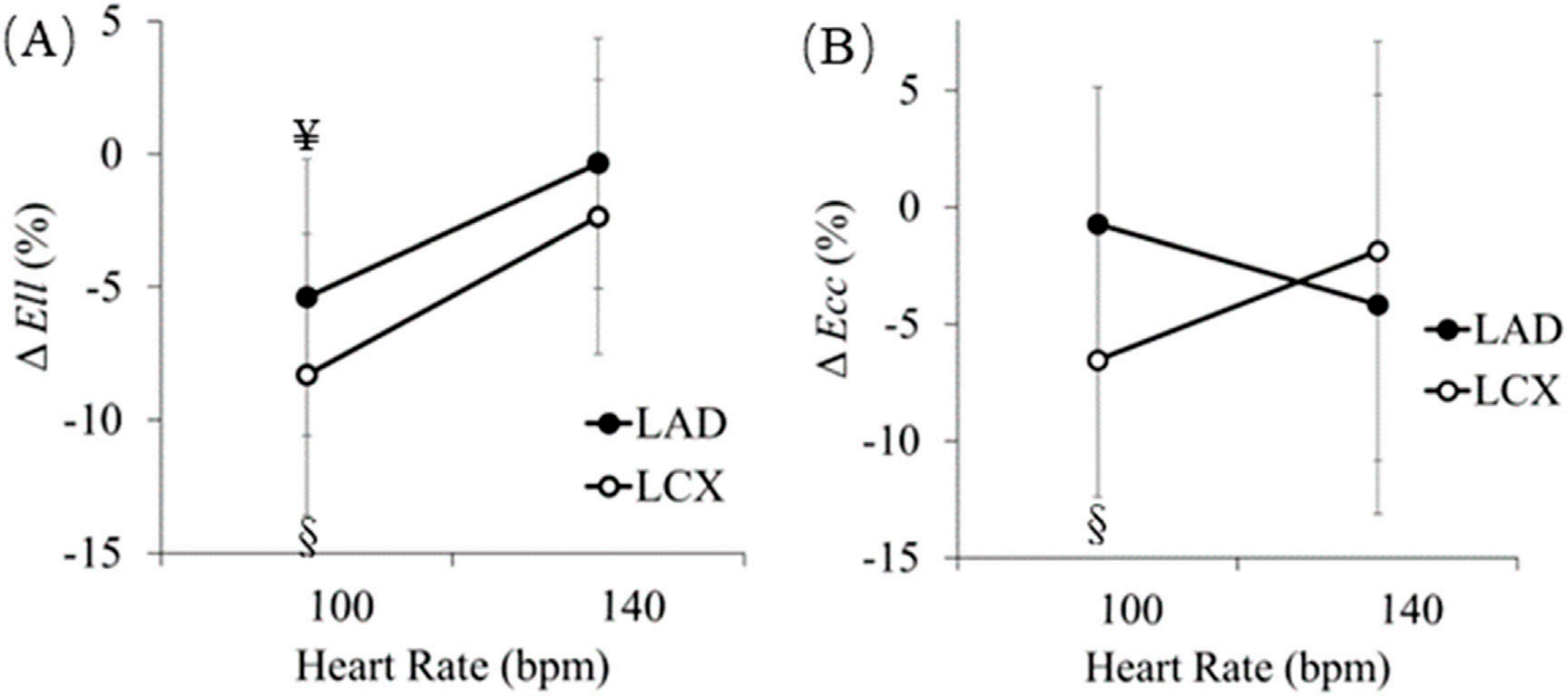
Differences between right ventricular pacing (RVP) and right atrial pacing (RAP) (RVP-RAP) in regional **(A)** longitudinal and **(B)** circumferential strains in the septum and LVFW measured with from 3D ECHO images, at 100 bpm and 140 bpm. §*p* < 0.05, RAP vs. RVP ¥ *p* < 0.01, RAP vs. RVP.

Figure 7 shows the changes in peak value of regional longitudinal and circumferential strains (*E*_*ll*_ and *E*_*cc*_) in the regions perfused by the LAD and LCX territories, respectively, between RAP and RVP. As strain is negative, peak *E*_*ll*_ in both regions increase at both 100 bpm and 140 bpm (5.39% ± 5.19%, *p* < 0.05 associated with the LAD, and 8.31% ± 5.30%, *p* < 0.01 associated with the LCX at 100 bpm, and 0.31% ± 4.71% associated with the LAD, and 2.10% ± 5.17% associated with the LCX at 140 bpm) (Figure 7A). Similarly, peak *E*_*cc*_ in both regions increase at both 100 bpm and 140 bpm (0.73% ± 5.87%, associated with the LAD, and 6.55% ± 8.94%, *p* < 0.05 associated with the LCX at 100 bpm, and 4.16% ± 8.97% associated with the LAD, and 1.88% ± 6.45% associated with the LCX at 140 bpm) (Figure 7B).

**FIGURE 8:**
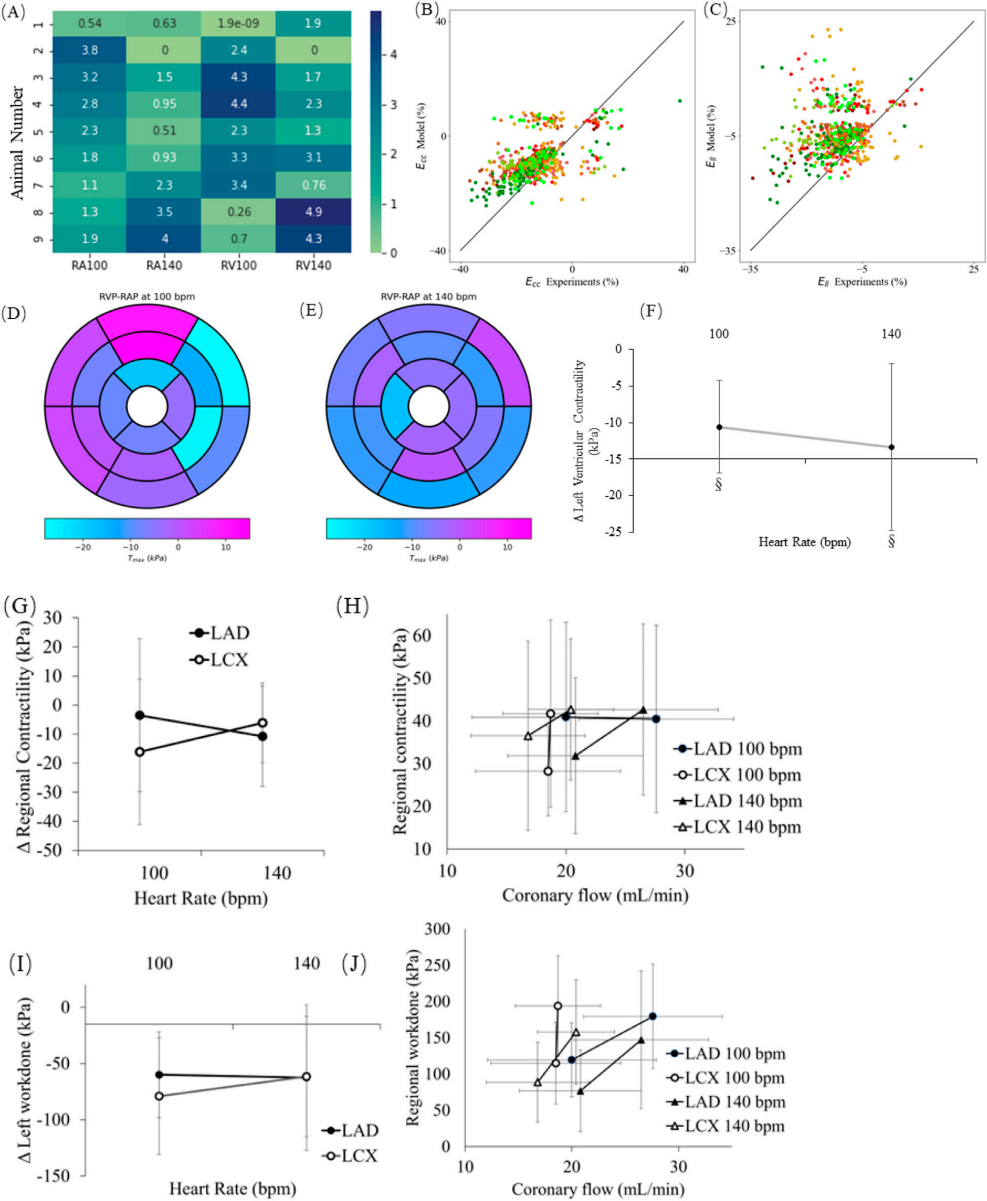
**(A)** The root mean square errors of model predicted and experimentally measured LVP under right atrial pacing (RAP) and right ventricular pacing (RVP) at 100 bpm and 140 bpm from 9 swine models are less than 5%. Correlations of model predicted and experimentally measured peak regional **(B)** circumferential strains and **(C)** longitudinal strains, in each AHA segment. Model predicted changes in regional contractility at **(D)** 100 bpm and **(E)** 140 bpm, during RAP and RVP. **(F)** Model predicted changes in global contractility during RAP and RVP. **(G)** Model predicted contractility changes between RAP and RVP in the regions where the LAD and LCX territories perfuse. **(H)** Correlation of experimentally measured regulated coronary flow in the LAD and LCX with model predicted regional contractility in the regions associated with the LAD and LCX. On each line, the point with higher coronary flow and contractility indicates RAP. **(I)** Model predicted work done changes between RAP and RVP in the regions where the LAD and LCX territories perfuse. **(J)** Correlation of experimentally measured regulated coronary flow in the LAD and LCX with model predicted regional work done in the regions associated with the LAD and LCX. On each line, the point with higher coronary flow and work done indicates RAP. §*p* < 0.05, RAP vs. RVP ¥ *p* < 0.01, RAP vs. RVP.

Figure 8 shows the inverse FE computational model predicted regional distribution of myocardial contractility. The root-mean square errors of model predicted and experimentally measured LVP during RAP and RVP at 100 bpm and 140 bpm from 9 swine models are less than 5% (Figure 8A). The overall fit of the regional circumferential and longitudinal strains is consistent with our previous study (Finsberg et al., 2019), where the model prediction shows a positive correlation with the experimental measurements (Figures 8B,C). The computational model predictions show that contractility is reduced in most regions with RVP except at the basal and midwall posterior regions when paced at 100 bpm (Figures 8D,E). The model predicted overall contractility reduces between RAP and RVP at both 100 bpm and 140 bpm by 10.6 ± 6.4 kPa, *p* < 0.05 and 13.4 ± 11.5 kPa, *p* < 0.05, respectively (Figure 8F). Contractility in regions perfused by the LAD is relatively unchanged at 100 bpm but is reduced at 140 bpm (10.8 ± 17.2 kPa) with RVP compared to RAP. Contractility in regions perfused by the LCX is reduced with RVP (13.5 ± 25.3 kPa at 100 bpm and 6.2 ± 13.7 kPa at 140 bpm) (Figure 8G). The model estimated regional contractility in regions perfused by the LAD and LCX is positively correlated with the corresponding measured regulated coronary flow rates. The gradient of the correlation, however, varies by region and heart rate (Figure 8H). The gradient associated with the LCX territory (67.35 kPa · min/mL) is significantly higher than that of the LAD (0) at 100 bpm but the gradient of the LCX territory (1.71 kPa · min/mL) is slightly lower than that of the LAD (1.89 kPa · min/mL) at 140 bpm. Regional myocardial work done estimated from the inverse FE computational modeling is reduced with RVP (59.9 ± 38.2 kPa at 100 bpm and 62.5 ± 64.7 kPa at 140 bpm associated with the LAD territory, and 79.1 ± 51.8 kPa at 100 bpm and 61.5 ± 53.5 kPa at 140 bpm associated with the LCX territory) (Figure 8I), and they are positively correlated with the corresponding regulated coronary flow rates in the LAD and LCX (Figure 8J). The gradient of the correlation, however, varies by region and heart rate (Figure 8J). The gradient associated with the LCX territory (395.30 kPa · min/mL) is significantly higher than that of the LAD (7.89 kPa · min/mL) at 100 bpm and the gradient of the LCX territory (19.22 kPa · min/mL) is higher than that of the LAD (12.33 kPa · min/mL) at 140 bpm.

## Discussion

In the present study, RVP caused mechanical dyssynchrony and acute changes in the LV function, hemodynamics and the coronary blood flow. Experimental measurements show that mechanical dyssynchrony resulted in significant decrease in LVESP (∼25–35 mmHg), dP/dt_(max)_ (∼400 mmHg/s), absolute dP/dt_(min)_ (∼400–900 mmHg/s), stroke volume (∼7–10 mL), cardiac output (∼985–1010 mL/min), regional longitudinal strain in the septum (∼0.3–5.4%) and LVFW (∼2.1–8.3%), circumferential strain in the septum (∼0.7–4.2%) and LVFW (∼1.9–6.6%), and the mean flow rate in the LAD under both regulated (∼6–8 mL/min) and passive (∼33–54 mL/min) conditions. On the contrary, the mean LCX regulated flow rate did not change, whereas the mean LCX passive flow rate decreased significantly (∼18–31 mL/min) with mechanical dyssynchrony. CFR is significantly decreased in both the LAD and LCX arteries at 140 bpm (∼1.6–1.8). By integrating the animal-specific LV geometry based on 3D ECHO images and measurements in the inverse FE computational model, the model shows a reduction in global contractility (∼79.3–100.2 mmHg) and regional contractility (∼16.2–93.3 mmHg in septum and ∼46.2–121.0 mmHg in the LVFW) with mechanical dyssynchrony. These reductions are positively correlated with the corresponding regulated coronary flow rates in the LAD and LCX. In addition, the model predicted regional myocardial work done reduced in the septum (∼59.9–62.5 kPa) and LVFW (∼61.5–79.1 kPa), which is positively correlated with the reduction in regulated coronary flow rates in the LAD and LCX. Overall, the findings demonstrate that effects of mechanical dyssynchrony on regional LV contractile function and myocardial work done correlate with the changes of coronary flow. This interrelated mechanism may play an important role in affecting CRT responder rate.

Animal models of mechanical dyssynchrony with RVP have been used to study the disease effects, classify patients’ risk, select the best treatment options, and anticipate the prognosis in heart failure patients (Zhang and Yu, 2012). Most animal studies, however, have focused mainly on the effects of mechanical dyssynchrony on LV function and hemodynamics. Specifically, the detrimental effects of mechanical dyssynchrony have been largely demonstrated in dogs (Spragg et al., 2003; Chakir et al., 2008; Prinzen et al., 1990; Beppu et al., 1997; Prinzen et al., 1999) and swine (Abd-Elmoniem et al., 2012; Rigol et al., 2013; Duchat et al., 2014; Zhou et al., 2015). The abnormal contraction pattern associated with mechanical dyssynchrony has been shown in these animal studies to lead to a reduction in LVESP, LV twist, and dP/dt_(max)_ (Zhou et al., 2015). Our findings are on par with these animal studies and are also consistent with clinical observations. For example, Lieberman and colleagues (Lieberman et al., 2006) found that in patients with EF >40%, RVP caused a decrease in stroke volume, cardiac output, EF, and dP/dt_(min)_, whereas in patients with EF <40% the effects were more pronounced and also affected LVESP and dP/dt_(max)_. Our results showed that RVP produces similar detrimental effects on LV hemodynamics and function. However, most previous studies demonstrated the effects of mechanical dyssynchrony on global LV contractile function based on dP/dt_(max)_. In this work, the effects of mechanical dyssynchrony on regional longitudinal strain and circumferential strains have also been analyzed, where regional longitudinal strain was reduced by ∼0.3–5.4% in the septum and by ∼2.1–8.3% in the LVFW, respectively. The reduction in circumferential strain is ∼0.7–4.2% in the septum and ∼1.9–6.6% in the LVFW. Previous study reported that the global longitudinal strain is reduced with LBBB by 13% (De Boeck et al., 2008). Furthermore, an inverse FE computational modeling framework has been developed based on these animal-specific measurements of regional strains and LV PV loops, and 3D ECHO images to predict the effects of mechanical dyssynchrony on regional contractility. The results show that mechanical dyssynchrony is associated with a reduction in global contractility (∼10.57–13.36 kPa at 100 bpm and 140 bpm) and regional contractility in the septum (∼2.16–12.43 kPa at 100 bpm and 140 bpm) and the LVFW (∼6.15–16.12 kPa at 100 bpm and 140 bpm) (Figure 8).

Due to the strong interaction between the myocardium and coronary vasculature (Westerhof et al., 2006), abnormal contraction in the LV may also lead to changes in coronary perfusion, which in turn, can further affect global LV function (Fang et al., 2013). The importance of coronary perfusion in mechanical dyssynchrony is also underscored in several recent studies, which suggest that the preservation of coronary blood flow may be one of the underlying mechanisms associated with a better outcome in CRT responders (Dik et al., 2014; Itoh et al., 2015; Yufu et al., 2019). The effects of mechanical dyssynchrony on coronary flow, however, are less studied in animal models, which can distinguish between the chronic (involving remodeling) and acute effects that most human studies are unable to do. Moreover, most animal studies do not investigate the impact of mechanical dyssynchrony on coronary flow under passive conditions as well as CFR. Most animal studies are also performed on canines (Prinzen et al., 1990; Ono et al., 1992; Amitzur et al., 1995; Beppu et al., 1997), which have marked differences in their coronary anatomy compared to humans (Lelovas et al., 2014). For example, the dominant source of blood supply to the canine myocardium is via the LCX artery whereas the LAD artery is usually the dominant source in swine and humans (Blair, 1961). The present study addresses these limitations by investigating the acute effects of mechanical dyssynchrony on coronary flow under both regulated and passive conditions in a translational animal model, with a coronary anatomy and perfusion distribution of blood flow like humans. This is significant because passive coronary flow, or pharmacologically vasodilated flow, is commonly assessed in clinical settings to identify factors affecting coronary flow besides those related to microvascular dysfunction of coronary flow regulation (Mehta et al., 2022), a key determinant of CFR. Our findings that passive coronary flow was reduced in the LAD and LCX suggest that impaired CFR was partially caused by changes in perfusion pressure or extravascular pressure (IMP) associated with mechanical dyssynchrony other than microvascular remodeling. Therefore, our findings have direct translational relevance by highlighting potential mechanisms that can limit CFR in pathophysiological states analogous to those encountered in patient care.

Consistent with findings in the canine model of mechanical dyssynchrony (Prinzen et al., 1990; Ono et al., 1992; Amitzur et al., 1995; Beppu et al., 1997), regulated blood flow in the early activated LAD region was significantly reduced compared to that in the late activated LCX region. A significant reduction in LAD blood flow under passive (vasodilated) conditions was also found in our study. The effects of mechanical dyssynchrony on the LCX blood flow are less consistent, with some studies reporting an increase in blood flow (Prinzen et al., 1990) and others reporting no change (Ono et al., 1992; Amitzur et al., 1995) in the late activated region. Our results agree with the latter and we further show that the LCX blood flow is reduced in mechanical dyssynchrony under passive conditions. We note that studies reporting an increase in LCX blood flow are conducted on canine model, which has substantial collaterals compared to swine model. Due to the changes in passive and regulated flow, CFR was reduced in both the LAD and LCX arteries at a high pacing rate (140 bpm) in our study. This rate-dependent reduction in CFR becoming significant at higher pacing rate of 140 bpm is likely due to shortened diastolic time, which reduces the time duration of coronary perfusion. This effect is particularly pronounced in territories with reduced baseline CFR, where regulation capacity is nearly exhausted at resting conditions. Besides, elevated IMP at higher heart rate may further impede blood flow. Clinically, these findings suggest that in patients with atrial fibrillation (Miyasaka et al., 2007) and rapid ventricular response (Brookes et al., 1999) may be at increased risk of ischemia, especially in territories affected by regional microvascular dysfunction. These results are also consistent with clinical observations showing hypoperfusion in the LV septum (Kyriakides et al., 2007) and a lower LAD CFR in LBBB patients with normal coronary arteries (Skalidis et al., 1999), suggesting that the acute effects of mechanical dyssynchrony may contribute (at least in part) to some of these features found in patients. This postulation has also been corroborated in some clinical studies showing that the correction of mechanical dyssynchrony by CRT improves coronary flow acutely (Claridge et al., 2015) and reduces microvascular resistance in a relatively short period of time (Kyriakides et al., 2007). The changes in coronary flow have been attributed to a redistribution in myocardial work and oxygen demand associated with mechanical dyssynchrony, with an increase (and decrease) in myofiber work in the late (and early) activated regions (Prinzen et al., 1990; Prinzen et al., 1999). This reasoning, however, cannot explain the changes in passive flow found here and other studies because the coronary vessels cannot regulate blood flow based on metabolic demand under vasodilated conditions. Given the close interaction between myocardium and coronary circulation, it is also difficult to isolate the different confounding factors responsible for these changes.

To explain these results and better understand the mechanisms behind mechanical dyssynchrony, our group has developed an experimentally-calibrated closed-loop cardiac-coronary computational model that considers the interactions between LV mechanics, systemic circulation, and coronary perfusion (Fan et al., 2020; Fan et al., 2021c). Cardiac-coronary interactions occur via three distinct mechanisms, namely, perfusion pressure generated by the LV, intramyocardial pressure (IMP, extravascular forces) in the myocardium, and contractility-coronary flow relationship. We have shown that the changes in passive coronary flow associated with mechanical dyssynchrony can be attributed to the combined effects of a reduction in perfusion pressure, an increase in IMP in the LAD territory, and a reduction in IMP in the LCX territory that altogether, can reproduce our findings in LAD and LCX passive flow. Since passive flow excludes the effects of coronary flow regulation, changes in the passive coronary flow in the LAD and LCX are attributed to perfusion pressure and regional IMP that varies regionally. Furthermore, although it is experimentally or clinically challenging to measure regional IMP, our developed computational model is able to predict regional IMP, which is a key determinant to the observed empirical territorial patterns. These results are also consistent with the changes in septal IMP measured in a few studies (Ono et al., 1992; Kaźmierczak et al., 2014). In a subsequent study (Fan et al., 2021c), we also showed that the significant reduction in LVEDP and LVESP (Figure 1A) as well as dP/dt_(max)_ and dP/dt_(min)_ (Figure 1B) with RVP can be explained by a reduction in coronary flow which decreases global LV contractility, and hence, may further reduce coronary perfusion pressure and flow.

Based on the computational modeling and experiments, we conclude that the adverse effects of mechanical dyssynchrony on LV function and coronary hemodynamics are likely not features occurring in isolation but instead, are interrelated with one another. Specifically, the coronary flow rate in the LAD and LCX shows positive correlations with regional contractility in the regions associated with each coronary network but with different gradients at different regions and heart rate (Figure 8H). The correlation is consistent with the previously proposed “perfusion-contraction matching” (Ross, 1991). The positive correlation was also found between model predicted myocardial work done and measured regulation coronary flow in the LAD and the LCX (Figure 8J), which can explain the “myocardial supply/demand imbalance” (Heusch, 2019; Fan et al., 2024).

In this study, mechanical dyssynchrony was induced by RVP, which affects both electromechanical and coronary functions. In contrast to RVP, which depolarizes the ventricles through slow conduction and induces LBBB, conduction-system pacing (CSP) (Jastrzebski et al., 2023) including His bundle pacing (HBP) and left bundle branch area pacing (LBBAP) maintains a near-normal QRS duration and synchronous mechanical activation. Clinical studies have demonstrated that CSP can prevent pacing-induced LV dysfunction and improve outcomes compared with conventional RVP, and in some cases provide benefits comparable to or exceeding CRT in patients with conduction disease (Pujol-lopez et al., 2022). These properties suggest that CSP may attenuate the adverse electromechanical and perfusion effects induced by RVP, particularly in structurally normal hearts, supporting translational and clinical evaluation of CSP as a physiological alternative to RVP in scenarios requiring chronic pacing (Edvardsen et al., 2024). It should also be noted that RAP (DDD) preserves atrioventricular (AV) synchrony, whereas RVP (VVI) does not. Thus, some of the hemodynamic differences may be related to the loss of atrial contribution in addition to ventricular mechanical dyssynchrony.

### Study limitations

We studied the effects of mechanical dyssynchrony in normal hearts. Although RVP-induced mechanical dyssynchrony has been used in prior studies as a surrogate of LBBB, demonstrating the hallmark feature of delayed activation of the LV lateral wall (Little et al., 1982; Kingma et al., 1983; Dohi et al., 2006), changes in function, hemodynamics and contraction with normal conduction may differ from those in failing hearts with LBBB. Ghani et al. (2011) for example, have reported differences in the mechanical activation pattern of the LV between both entities, which agree with findings (differences in electromechanical delay, contraction and relaxation times, as well as uncoordinated LV wall motion) by Xiao et al. (1993). Witte and collaborators (Witte et al., 2006), however, found similar dyssynchrony in patients with RVP-induced LBBB and patients with intrinsic LBBB. In structurally normal hearts, RVP-induced delayed activation occurs in a preserved Purkinje fiber network, normal myocardium and contractility, producing acute but potentially reversible dyssynchrony. By contrast, intrinsic LBBB in failing hearts is often accompanied by conduction system degeneration, regional fibrosis, or LV dilation, accelerating mechanical dyssynchrony (Kanawati and Sy, 2018). These differences between normal and failing hearts may alter the magnitude of changes in LV and coronary function. In heart failure, the presence of microvascular dysfunction may exacerbate the impairment of CFR as found in our study. Thus, our findings isolate the hemodynamic consequences of conduction delay in the absence of structural disease, representing an upper bound of physiological effects, and clinical extrapolation to failing hearts should be made with these distinctions. Future studies in animal models of ischemic heart failure and structural defects will address these differences. The differences in animal models, however, do not alter the major conclusion of our study. Furthermore, the pacing rates used in this study (100 and 140 bpm) are higher than typical clinical pacemaker settings in pacemaker-dependent patients at rest (50–70 bpm). These elevated rates were intentionally selected to ensure consistency and to allow direct rate comparisons across animals. While this design improves experimental control and highlights rate-dependent physiological effects, it may limit the direct extrapolation of our findings to resting patients with chronic pacing. In future studies, pacing at resting heart rates will be considered. Finally, the pacing modes (RAP vs. RVP) compared differ in AV synchrony, where RAP (DDD) maintains AV synchrony, whereas RVP (VVI) does not. Therefore, the hemodynamic differences observed may reflect both ventricular dyssynchrony and the absence of atrial contribution during VVI pacing.

## Conclusion

We have performed experimental measurements in a clinically relevant swine model with coronary anatomy and perfusion characteristics resembling that of humans, and developed an animal-specific inverse finite element computational model to investigate the acute effects of mechanical dyssynchrony on LV hemodynamics and function as well as coronary perfusion. We show that mechanical dyssynchrony not only negatively affects the global LV function and hemodynamics (e.g., SDI, LVESP, LVEF, cardiac output, and global myocardial strain), and regional LV function (e.g., regional myocardial strain, model predicted contractility and myocardial work done), but also reduces LAD coronary blood flow rate under both passive and regulated conditions, LCX coronary flow rate under passive condition and CFR at higher heart rates. The adverse effects of mechanical dyssynchrony on regional LV function (e.g., regional contractility and myocardial work done) and coronary hemodynamics show positive correlations in septal and LVFW regions. These findings demonstrate that these interrelated factors of the regional LV contractility, myocardial work done, and coronary flow may have clinical implications in the identification of CRT non-responders and improvement in treatment strategies.

## Data Availability

The original contributions presented in the study are included in the article/supplementary material, further inquiries can be directed to the corresponding author.

## Abbreviations

HF, Heart failure; CRT, Cardiac resynchronization therapy; LV, Left ventricle/ventricular; LBBB, Left bundle branch block; SDI, Systolic dyssynchrony index; BL, Baseline; RAP, Right atrial pacing; RVP, Right ventricular pacing; LVEDP, Left ventricular end-diastolic pressure; LVESP, Left ventricular end-systolic pressure; LVEDV, Left ventricular end-diastolic volume; LVESV, Left ventricular end-systolic volume; EF, Ejection fraction; CFR, Coronary flow reserve; LAD, Left anterior descending; LCX, Left circumflex; LVFW, Left ventricular free wall; FE, Finite element; IMP, Intramyocardial pressure.

## References

[B1] Abd-Elmoniem K. Z. Tomas M. S. Sasano T. Soleimanifard S. Vonken E. J. P. Youssef A. (2012). Assessment of distribution and evolution of Mechanical dyssynchrony in a porcine model of myocardial infarction by cardiovascular magnetic resonance. J. Cardiovasc Magn. Reson 14, 1–10. 10.1186/1532-429X-14-1 22226320 PMC3268109

[B2] Amitzur G. Manor D. Pressman A. Adam D. Hammerman H. Shofti R. (1995). Modulation of the arterial coronary blood flow by asynchronous activation with ventricular pacing. Pacing Clin. Electrophysiol. 18, 697–710. 10.1111/j.1540-8159.1995.tb04664.x 7596853

[B3] Beppu S. Matsuda H. Shishido T. Miyatake K. (1997). Functional myocardial perfusion abnormality induced by left ventricular asynchronous contraction: experimental study using myocardial contrast echocardiography. J. Am. Coll. Cardiol. 29, 1632–1638. 10.1016/S0735-1097(97)82542-9 9180129

[B4] Blair E. (1961). Anatomy of the ventricular coronary arteries in the dog. Circ. Res. 9, 333–341. 10.1161/01.res.9.2.333

[B5] Boriani G. Nesti M. Ziacchi M. Padeletti L. (2015). Cardiac resynchronization therapy: an overview on guidelines. Card. Electrophysiol. Clin. 7, 673–693. 10.1016/j.ccep.2015.08.015 26596811

[B6] Brookes C. Ravn H. White P. Moeldrup U. Oldershaw P. Redington A. (1999). Acute right ventricular dilatation in response to ischemia significantly impairs left ventricular systolic performance. Circulation 100, 761–767. 10.1161/01.CIR.100.7.761 10449700

[B7] Cazeau S. Toulemont M. Ritter P. Reygner J. (2019). Statistical ranking of electromechanical dyssynchrony parameters for CRT. Open hear. 6, e000933–e000939. 10.1136/openhrt-2018-000933 30740229 PMC6347881

[B8] Chakir K. Daya S. K. Tunin R. S. Helm R. H. Byrne M. J. Dimaano V. L. (2008). Reversal of global apoptosis and regional stress kinase activation by cardiac resynchronization. Circulation 117, 1369–1377. 10.1161/CIRCULATIONAHA.107.706291 18316490

[B9] Claridge S. Chen Z. Jackson T. De Silva K. Behar J. Sohal M. (2015). Effects of epicardial and endocardial cardiac resynchronization therapy on coronary flow: insights from wave intensity analysis. J. Am. Heart Assoc. 4, e002626–12. 10.1161/JAHA.115.002626 26679935 PMC4845290

[B10] Cortigiani L. Rigo F. Gherardi S. Bovenzi F. Molinaro S. Picano E. (2013). Prognostic implication of Doppler echocardiographic derived coronary flow reserve in patients with left bundle branch block. Eur. Heart J. 34, 364–373. 10.1093/eurheartj/ehs310 23008505

[B11] De Boeck B. W. L. Kirn B. Teske A. J. Hummeling R. W. Doevendans P. A. Cramer M. J. (2008). Three-dimensional mapping of mechanical activation patterns, contractile dyssynchrony and dyscoordination by two-dimensional strain echocardiography: rationale and design of a novel software toolbox. Cardiovasc Ultrasound 6, 22–12. 10.1186/1476-7120-6-22 18513412 PMC2429897

[B12] Dikic A. D. Nikcevic G. Raspopovic S. Jovanovic V. Tesic M. Beleslin B. (2014). Prognostic role of coronary flow reserve for left ventricular functional improvement after cardiac resynchronization therapy in patients with dilated cardiomyopathy. Eur. Heart J. Cardiovasc Imaging 15, 1344–1349. 10.1093/ehjci/jeu136 25053732

[B13] Dohi K. Pinsky M. R. Kanzaki H. Severyn D. Gorcsan J. (2006). Effects of radial left ventricular dyssynchrony on cardiac performance using quantitative tissue Doppler radial strain imaging. J. Am. Soc. Echocardiogr. 19, 475–482. 10.1016/j.echo.2005.10.017 16644429

[B14] Duchateau N. Sitges M. Doltra A. Fernández-Armenta J. Solanes N. Rigol M. (2014). Myocardial motion and deformation patterns in an experimental swine model of acute LBBB/CRT and chronic infarct. Int. J. Cardiovasc Imaging 30, 875–887. 10.1007/s10554-014-0403-2 24651923

[B15] Edvardsen T. Ross S. Kongsgård E. (2024). Enhancing cardiac pacing strategies: a review of conduction system pacing compared with right and biventricular pacing and their influence on myocardial function. Eur. Hear J. - Cardiovasc Imaging 25, 879–887. 10.1093/ehjci/jeae090 PMC11210972 38565632

[B16] Fan L. Namani R. Choy S. Kassab G. S. Lee L. C. (2020). Effects of mechanical dyssynchrony on coronary flow: insights from a computational model of coupled coronary perfusion with systemic circulation. Front. Physiol. 11, 915. 10.3389/fphys.2020.00915 32922304 PMC7457036

[B17] Fan L. Choy J. S. Raissi F. Kassab G. S. Lee L. C. (2021a). Optimization of cardiac resynchronization therapy based on a cardiac electromechanics-perfusion computational model. Comput. Biol. Med. 141, 105050. 10.1016/j.compbiomed.2021.105050 34823858 PMC8810745

[B18] Fan L. Namani R. Choy J. S. Kassab G. S. Lee L. C. (2021b). Transmural distribution of coronary perfusion and myocardial work density due to alterations in ventricular loading, geometry and contractility. Front. Physiol. 12, 744855–20. 10.3389/fphys.2021.744855 34899378 PMC8652301

[B19] Fan L. Namani R. Choy J. S. Awakeem Y. Kassab G. S. Lee L. C. (2021c). Role of coronary flow regulation and cardiac-coronary coupling in mechanical dyssynchrony associated with right ventricular pacing. Am. J. Physiol. - Hear Circ. Physiol.;320: H1037–H1054. 10.1152/ajpheart.00549.2020 33356963 PMC8294701

[B20] Fan L. Choy J. S. Lee S. Campbell K. S. Wenk J. F. Kassab G. S. (2023). An *in silico* study of the effects of left ventricular assist device on right ventricular function and inter-ventricular interaction. Artif. Organs 47, 1831–1847. 10.1111/aor.14649 37746896 PMC10964177

[B21] Fan L. Wang H. Kassab G. Lee L. C. (2024). Review of cardiac – coronary interaction and insights from mathematical modeling. WIREs Mech. Dis. 16, e1642–37. 10.1002/wsbm.1642 38316634 PMC11081852

[B22] Fang F. Chan J. Y. S. Lee A. P. W. Sung S. H. Luo X. X. Jiang X. (2013). Improved coronary artery blood flow following the correction of systolic dyssynchrony with cardiac resynchronization therapy. Int. J. Cardiol. 167, 2167–2171. 10.1016/j.ijcard.2012.05.094 22704862

[B23] Finsberg H. Xi C. Tan J. Le Zhong L. Genet M. Sundnes J. (2018). Efficient estimation of personalized biventricular mechanical function employing gradient-based optimization. Int. J. Numer. method Biomed. Eng. 34, e2982. 10.1002/cnm.2982 29521015 PMC6043386

[B24] Finsberg H. Xi C. Zhao X. Tan J. Le Genet M. Sundnes J. (2019). Computational quantification of patient-specific changes in ventricular dynamics associated with pulmonary hypertension. Am. J. Physiol. - Hear Circ. Physiol. 317, H1363–H1375. 10.1152/AJPHEART.00094.2019 31674809 PMC7132315

[B25] Ghani A. Delnoy PPHM Ottervanger J. P. Ramdat Misier A. R. Smit J. J. J. Elvan A. (2011). Assessment of left ventricular dyssynchrony in pacing-induced left bundle branch block compared with intrinsic left bundle branch block. Europace 13, 1504–1507. 10.1093/europace/eur117 21527389

[B26] Gorcsan J. (2011). Finding pieces of the puzzle of nonresponse to cardiac resynchronization therapy. Circulation 123, 10–12. 10.1161/CIRCULATIONAHA.110.001297 21173347

[B27] Gsell M. A. F. Augustin C. M. Prassl A. J. Karabelas E. Fernandes J. F. Kelm M. (2018). Assessment of wall stresses and mechanical heart power in the left ventricle: finite element modeling versus Laplace analysis. Int. J. Numer. method Biomed. Eng. 34, e3147–18. 10.1002/cnm.3147 30151998 PMC6492182

[B28] Harb S. C. Toro S. Bullen J. A. Obuchowski N. A. Xu B. Trulock K. M. (2019). Scar burden is an independent and incremental predictor of cardiac resynchronisation therapy response. Open hear. 6, e001067–14. 10.1136/openhrt-2019-001067 31354957 PMC6615837

[B29] Heusch G. (2019). Myocardial ischemia: lack of coronary blood flow, myocardial oxygen supply-demand imbalance, or what? Am. J. Physiol. - Hear Circ. Physiol. 316, H1439–H1446. 10.1152/ajpheart.00139.2019 31002282 PMC7137753

[B30] Itoh M. Shinke T. Yoshida A. Kozuki A. Takei A. Fukuzawa K. (2015). Reduction in coronary microvascular resistance through cardiac resynchronization and its impact on chronic reverse remodelling of left ventricle in patients with non-ischaemic cardiomyopathy. Europace 17, 1407–1414. 10.1093/europace/euu361 25662988

[B31] Jastrzebski M. Dandamudi G. Burri H. Ellenbogen K. A. (2023). Conduction system pacing: overview, definitions, and nomenclature. Eur. Hear J. Suppl. 25, 4–14. 10.1093/eurheartjsupp/suad114 37970514 PMC10637837

[B32] Kanawati J. Sy R. W. (2018). Contemporary review of left bundle branch block in the failing heart – pathogenesis, prognosis, and therapy. Hear Lung Circ. 27, 291–300. 10.1016/j.hlc.2017.09.007 29097067

[B33] Kaźmierczak J. Peregud-Pogorzelska M. Gora̧cy J. Wojtarowicz A. Kiedrowicz R. Kornacewicz-Jach Z. (2014). Effect of cardiac resynchronisation therapy on coronary blood flow in patients with non-ischaemic dilated cardiomyopathy. Kardiol. Pol. 72, 511–518. 10.5603/KP.a2014.0019 24526554

[B34] Kerckhoffs R. C. P. Faris O. P. Bovendeerd P. H. M. Prinzen F. W. Smits K. McVeigh E. R. (2005). Electromechanics of paced left ventricle simulated by straightforward mathematical model: comparison with experiments. Am. J. Physiol. - Hear Circ. Physiol. 289, 1889–1897. 10.1152/ajpheart.00340.2005 15964924 PMC2396318

[B35] Kingma I. Tyberg J. V. Smith E. R. (1983). Effects of diastolic transseptal pressure gradient on ventricular septal position and motion. Circulation 68, 1304–1314. 10.1161/01.CIR.68.6.1304 6640880

[B36] Kirk J. A. Kass D. A. (2013). Electromechanical dyssynchrony and resynchronization of the failing heart. Circ. Res. 113, 765–776. 10.1161/CIRCRESAHA.113.300270 23989718 PMC3874431

[B37] Kyriakides Z. S. Manolis A. G. Kolettis T. M. (2007). The effects of ventricular asynchrony on myocardial perfusion. Int. J. Cardiol. 119, 3–9. 10.1016/j.ijcard.2006.03.091 17056140

[B38] Lee A. W. C. Costa C. M. Strocchi M. Rinaldi C. A. Niederer S. A. (2018). Computational modeling for cardiac resynchronization therapy. J. Cardiovasc. Transl. Res. 11, 92–108. 10.1007/s12265-017-9779-4 29327314 PMC5908824

[B39] Lelovas P. P. Kostomitsopoulos N. G. Xanthos T. T. (2014). A comparative anatomic and physiologic overview of the porcine heart. J. Am. Assoc. Lab. Anim. Sci. 53, 432–438. 25255064 PMC4181683

[B40] Lieberman R. Padeletti L. Schreuder J. Jackson K. Michelucci A. Colella A. (2006). Ventricular pacing lead location alters systemic hemodynamics and left ventricular function in patients with and without reduced ejection fraction. J. Am. Coll. Cardiol. 48, 1634–1641. 10.1016/j.jacc.2006.04.099 17045900

[B41] Little W. C. Reeves R. C. Arciniegas J. Katholi R. E. Rogers E. W. (1982). Mechanism of abnormal interventricular septal motion. During delayed left ventricular activation. Circulation 65, 1486–1491. 10.1161/01.CIR.65.7.1486 7074805

[B42] Mehta P. K. Quesada O. Al-badri A. Fleg J. L. Santos A. Pepine C. J. (2022). Ischemia and no obstructive coronary arteries in patients with stable ischemic heart disease. Int. J. Cardiol. 348, 1–8. 10.1016/j.ijcard.2021.12.013 34902504 PMC8779638

[B43] Miyasaka Y. Barnes M. E. Gersh B. J. Cha S. S. Bailey K. R. Seward J. B. (2007). Coronary ischemic events after first atrial fibrillation: risk and survival. 120; 357–363.e1. 10.1016/j.amjmed.2006.06.042 17398231

[B44] Mojumder J. Fan L. Nguyen T. Campbell K. S. Wenk J. F. Guccione J. M. (2023). Computational analysis of ventricular mechanics in hypertrophic cardiomyopathy patients. Sci. Rep. 13, 958–17. 10.1038/s41598-023-28037-w 36653468 PMC9849405

[B45] Monge Garcia M. I. Jian Z. Settels J. J. Hunley C. Cecconi M. Hatib F. (2018). Performance comparison of ventricular and arterial dP/dtmax for assessing left ventricular systolic function during different experimental loading and contractile conditions. Crit. Care 22, 325–12. 10.1186/s13054-018-2260-1 30486866 PMC6262953

[B46] Murín P. Mitro P. Valocik G. Spurný P. (2015). Global myocardial contractile reserve assessed by high-dose dobutamine stress echocardiography predicts response to the cardiac resynchronization therapy. Echocardiography 32, 490–495. 10.1111/echo.12694 25059770

[B47] Normand C. Linde C. Singh J. Dickstein K. (2018). Indications for cardiac resynchronization therapy: a comparison of the major international guidelines. JACC Hear Fail 6, 308–316. 10.1016/j.jchf.2018.01.022 29598935

[B48] Ono S. Nohara R. Kambara H. Okuda K. Kawai C. (1992). Regional myocardial perfusion and glucose metabolism in experimental left bundle branch block. Circulation 85, 1125–1131. 10.1161/01.CIR.85.3.1125 1537110

[B49] Prinzen F. W. Augustijn C. H. Arts T. Allessie M. A. Reneman R. S. (1990). Redistribution of myocardial fiber strain and blood flow by asynchronous activation. Am. J. Physiol. - Hear Circ. Physiol. 259, H300–H308. 10.1152/ajpheart.1990.259.2.h300 2386214

[B50] Prinzen F. W. Hunter W. C. Wyman B. T. McVeigh E. R. (1999). Mapping of regional myocardial strain and work during ventricular pacing: experimental study using magnetic resonance imaging tagging. J. Am. Coll. Cardiol. 33, 1735–1742. 10.1016/S0735-1097(99)00068-6 10334450 PMC2041911

[B51] Pujol-lopez M. Garre P. Guasch E. Doltra A. Niebla M. Carro E. (2022). Conduction system pacing vs biventricular pacing in Heart Failure and wide QRS patients. Conduction Syst. Pacing vs Biventricular Pacing Heart Fail. Wide QRS Patients 8, 1431–1445. 10.1016/j.jacep.2022.08.001 36424012

[B52] Rigol M. Solanes N. Fernandez-Armenta J. Silva E. Doltra A. Duchateau N. (2013). Development of a swine model of left bundle branch block for experimental studies of cardiac resynchronization therapy. J. Cardiovasc Transl. Res. 6, 616–622. 10.1007/s12265-013-9464-1 23636845

[B53] Ross Jr J. (1991). Point of view myocardial perfusion-contraction matching implications for coronary heart disease and hibernation. Circulation 83, 1076–1084. 1999010 10.1161/01.cir.83.3.1076

[B54] Schwinger R. H. G. (2021). Pathophysiology of heart failure. Cardiovasc Diagn Ther. 11, 263–276. 10.21037/CDT-20-302 33708498 PMC7944197

[B55] Skalidis E. I. Kochiadakis G. E. Koukouraki S. I. Parthenakis F. I. Karkavitsas N. S. Vardas P. E. (1999). Phasic coronary flow pattern and flow reserve in patients with left bundle branch block and normal coronary arteries. J. Am. Coll. Cardiol. 33, 1338–1346. 10.1016/S0735-1097(98)00698-6 10193736

[B56] Solomon S. D. Foster E. Bourgoun M. Shah A. Viloria E. Brown M. W. (2010). Effect of cardiac resynchronization therapy on reverse remodeling and relation to outcome: multicenter automatic defibrillator implantation trial: cardiac resynchronization therapy. Circulation 122, 985–992. 10.1161/CIRCULATIONAHA.110.955039 20733097

[B57] Spragg D. D. Leclercq C. Loghmani M. Faris O. P. Tunin R. S. DiSilvestre D. (2003). “Regional alterations in protein expression in the dyssynchronous failing heart,”Circulation, 108. 929–932. 10.1161/01.cir.0000088782.99568.ca 12925451

[B58] Walmsley J. Arts T. Derval N. Bordachar P. Cochet H. Ploux S. (2015). Fast simulation of mechanical heterogeneity in the electrically asynchronous heart using the multipatch module. PLoS Comput. Biol. 11, e1004284–23. 10.1371/journal.pcbi.1004284 26204520 PMC4512705

[B59] Wang V. Y. Ennis D. B. Cowan B. R. Young A. A. Nash M. P. (2012). Myocardial contractility and regional work throughout the cardiac cycle using FEM and MRI. Lect. Notes Comput. Sci. Incl. Subser. Lect. Notes Artif. Intell. Lect. Notes Bioinforma. 7085 (LNCS), 149–159. 10.1007/978-3-642-28326-0_15

[B60] Wang V. Y. Nielsen P. M. F. Nash M. P. (2015). Image-based predictive modeling of heart mechanics. Annu. Rev. Biomed. Eng. 17, 351–383. 10.1146/annurev-bioeng-071114-040609 26643023

[B61] Westerhof N. Boer C. Lamberts R. R. Sipkema P. (2006). Cross-talk between cardiac muscle and coronary vasculature. Physiol. Rev. 86, 1263–1308. 10.1152/physrev.00029.2005 17015490

[B62] Witte K. K. A. Pipes R. R. Nanthakumar K. Parker J. D. (2006). Biventricular pacemaker upgrade in previously paced heart failure patients-improvements in ventricular dyssynchrony. J. Card. Fail 12, 199–204. 10.1016/j.cardfail.2005.12.003 16624685

[B63] Xiao H. B. Brecker S. J. D. Gibson D. G. (1993). Differing effects of right ventricular pacing and left bundle branch block on left ventricular function. Br. Heart J. 69, 166–173. 10.1136/hrt.69.2.166 8435243 PMC1024945

[B64] Yufu K. Kondo H. Shinohara T. Ishii Y. Yoshimura S. Abe I. (2019). Assessment of coronary flow reserve predicts long-term outcome of responders to cardiac resynchronization therapy. Heart Vessels 34, 763–770. 10.1007/s00380-018-1308-0 30483876

[B65] Zhang Q. Yu C. M. (2012). Clinical implication of mechanical dyssynchrony in heart failure. J. Cardiovasc Ultrasound 20, 117–123. 10.4250/jcu.2012.20.3.117 23185653 PMC3498307

[B66] Zhang N. Fan L. Choy J. S. Cai C. Teague S. D. Guccione J. (2024). Comparison of left ventricular function derived from subject-specific inverse finite element modeling based on 3D ECHO and magnetic resonance images. Bioeng 11, 735. 10.3390/bioengineering11070735 39061817 PMC11273843

[B67] Zhou W. Benharash P. Han Chua J. Nakahara S. Ho J. K. Mahajan A. (2015). Acute effects of pacing at different ventricular sites on left ventricular rotational mechanics in a porcine model. J. Cardiothorac. Vasc. Anesth. 29, 1148–1154. 10.1053/j.jvca.2014.12.008 25824449

[B68] Ziaeian B. Fonarow G. C. (2016). Epidemiology and aetiology of heart failure. Nat. Rev. Cardiol. 13, 368–378. 10.1038/nrcardio.2016.25 26935038 PMC4868779

